# 3-Methoxy­benzaldehyde thio­semi­carbazone

**DOI:** 10.1107/S160053680901040X

**Published:** 2009-03-28

**Authors:** Jian Zhang, Lin-ping Wu, Ling-hua Zhuang, Guo-wei Wang

**Affiliations:** a Department of Light Chemical Engineering, Nanjing University of Technology, Nanjing 210009, People’s Republic of China; b Department of Applied Chemistry, College of Science, Nanjing University of Technology, Nanjing 210009, People’s Republic of China

## Abstract

The title compound, C_9_H_11_N_3_OS, was prepared by the reaction of 3-methoxy­benzaldehyde and thio­semicarbazide. The benzyl­idene ring and the thio­semicarbazone fragment are slightly twisted, making a dihedral angle of 14.1 (1)°. A weak intra­molecular N—H⋯N hydrogen bond may influence the conformation of the mol­ecule. Inter­molecular N—H⋯S hydrogen bonds build up a three-dimensional network.

## Related literature

For a general background to thio­semicarbazone compounds, see: Casas *et al.* (2000 ▶); Tarafder *et al.* (2000 ▶); Ferrari *et al.* (2000 ▶); Deschamps *et al.* (2003 ▶); Maccioni *et al.* (2003 ▶); Chimenti *et al.*(2007 ▶). For bond-length data, see: Allen *et al.* (1987 ▶).
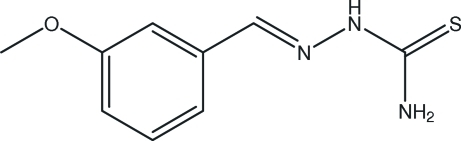


## Experimental

### 

#### Crystal data

C_9_H_11_N_3_OS
*M* *_r_* = 209.27
Monoclinic, 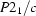

*a* = 11.814 (2) Å
*b* = 5.6760 (11) Å
*c* = 15.248 (3) Å
β = 90.29 (3)°
*V* = 1022.5 (3) Å^3^
*Z* = 4
Mo *K*α radiation
μ = 0.29 mm^−1^
*T* = 293 K
0.30 × 0.20 × 0.10 mm

#### Data collection

Enraf–Nonius CAD-4 diffractometer
Absorption correction: ψ scan (North *et al.*, 1968 ▶) *T*_min_ = 0.908, *T*_max_ = 0.969
1946 measured reflections
1852 independent reflections
1494 reflections with *I* > 2σ(*I*)
*R*_int_ = 0.017
3 standard reflections every 200 reflections intensity decay: 9%

#### Refinement

*R*[*F* ^2^ > 2σ(*F* ^2^)] = 0.041
*wR*(*F* ^2^) = 0.110
*S* = 1.06
1852 reflections
128 parameters
H-atom parameters constrained
Δρ_max_ = 0.20 e Å^−3^
Δρ_min_ = −0.26 e Å^−3^

### 

Data collection: *CAD-4 Software* (Enraf–Nonius, 1989 ▶); cell refinement: *CAD-4 Software*; data reduction: *XCAD4* (Harms & Wocadlo, 1995 ▶); program(s) used to solve structure: *SHELXS97* (Sheldrick, 2008 ▶); program(s) used to refine structure: *SHELXL97* (Sheldrick, 2008 ▶); molecular graphics: *SHELXTL* (Sheldrick, 2008 ▶) and *PLATON* (Spek, 2009 ▶); software used to prepare material for publication: *SHELXTL*.

## Supplementary Material

Crystal structure: contains datablocks global, I. DOI: 10.1107/S160053680901040X/dn2432sup1.cif

Structure factors: contains datablocks I. DOI: 10.1107/S160053680901040X/dn2432Isup2.hkl

Additional supplementary materials:  crystallographic information; 3D view; checkCIF report

## Figures and Tables

**Table 1:** Hydrogen-bond geometry (Å, °)

*D*—H⋯*A*	*D*—H	H⋯*A*	*D*⋯*A*	*D*—H⋯*A*
N2—H2⋯S1^i^	0.86	2.57	3.370 (2)	156
N3—H3*B*⋯S1^ii^	0.86	2.57	3.411 (2)	166
N3—H3*A*⋯N1	0.86	2.25	2.611 (3)	105

Symmetry codes: (i) 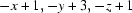; (ii) 
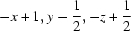
.

**Fig. 1.**
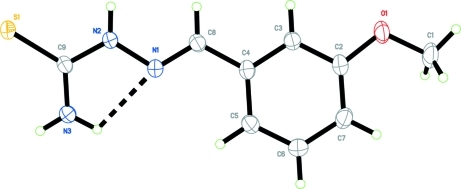
A view of the molecular structure of (I) showing the atom-numbering scheme and 30% displacement ellipsoids. H atoms are represented as smal sphere of arbitrary radii. Intramolecular hydrogen bond is shown as dashed line.

**Fig. 2.**
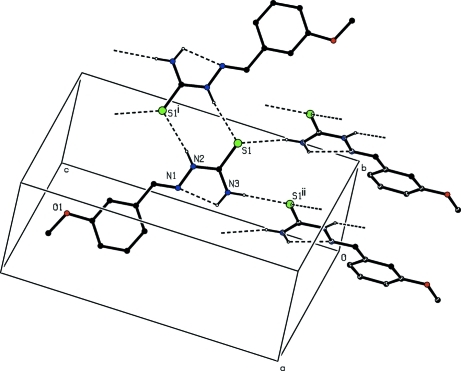
Partial packing view showing the N-H···S hydrogen bonds network. H atoms not involved in hydrogen bonding have been omitted for clarity. H bonds are shown as dashed lines. [Symmetry codes: (i) -x+1, -y+3, -z+1; (ii) -x+1, y-1/2, -z+1/2]

## supplementary crystallographic information

### Comment

Thiosemicarbazones constitute an important class of *N,S* donor ligands due to their propensity to react with a wide range of metals (Casas *et al.*, 2000). Thiosemicarbazones exhibit various biological activities and have therefore attracted considerable pharmaceutical interest (Maccioni *et al.*, 2003; Ferrari *et al.*, 2000). They have been evaluated as antiviral, antibacterial and anticancer therapeutics. Thiosemicarbazones belong to a large group of thiourea derivatives, whose biological activities are a function of parent aldehyde or ketone moiety (Chimenti *et al.*, 2007). Schiff bases show potential as antimicrobial and anticancer agents (Tarafder *et al.*, 2000; Deschamps *et al.*, 2003) and so have biochemical and pharmacological applications. We here report the crystal structure of the title compound (I).

The sulfur atom and the hydrazine nitrogen N1 are in *trans* position with respect to the C9–N2 bond. This conformation may be induced by the weak intramolecular N-H···N hydrogen bond (Fig. 1, Table 1). All bond lengths are within normal ranges (Allen *et al.*, 1987).

At first glance the molecule is roughly planar with the largest deviation from the mean plane being -0.272 (3) Å at N3, however the benzaldehyde ring and the thiosemicarbazone fragment are twisted with respect to each other making a dihedral angle of 14.1 (1)°.

The molecules are connected by intermolecular N—H···S hydrogen bonds which build up a three dimensional network (Table 1, Fig.2).

### Experimental

A mixture of 3-methoxybenzaldehyde (1.36 g, 0.01 mol) and hydrazinecarbothioamide (0.91 g, 0.01 mol) in 20 ml of absolute methanol was refluxed for about 3 h. On cooling, the solid separated was filtered and recrystallized from ethyl acetate. Crystals of (I) suitable for X-ray diffraction were obtained by slow evaporation of ethyl acetate. ^1^H NMR (DMSO, δ, p.p.m.) 11.39 (s, 1 H), 8.17 (s, 1 H), 8.02 (s,2 H), 7.42 (m, 1 H), 7.30 (t, 2 H), 6.99 (t,1 H), 3.79 (t, 3 H).

### Refinement

All H atoms were positioned geometrically, with C—H = 0.93 Å (aromatic) or 0.96 Å (methyl) and N—H = 0.86 Å, and constrained to ride on their parent atoms, with *U*_iso_(H) = *xU*_eq_(C), where *x*= 1.5 for methyl H and *x* = 1.2 for C(aromatic) and N atoms.

### Crystal data

**Table:**

C_9_H_11_N_3_OS	*F*(000) = 440
*M**_r_* = 209.27	*D*_x_ = 1.359 Mg m^−^^3^
Monoclinic, *P*2_1_/*c*	Mo *K*α radiation, λ = 0.71073 Å
Hall symbol: -P 2ybc	Cell parameters from 27 reflections
*a* = 11.814 (2) Å	θ = 1–25°
*b* = 5.6760 (11) Å	µ = 0.29 mm^−^^1^
*c* = 15.248 (3) Å	*T* = 293 K
β = 90.29 (3)°	Block, colorless
*V* = 1022.5 (3) Å^3^	0.30 × 0.20 × 0.10 mm
*Z* = 4	

### Data collection

**Table:**

Enraf–Nonius CAD-4 diffractometer	1494 reflections with *I* > 2σ(*I*)
Radiation source: fine-focus sealed tube	*R*_int_ = 0.017
graphite	θ_max_ = 25.3°, θ_min_ = 1.7°
ω/2θ scans	*h* = −14→0
Absorption correction: ψ scan (North *et al.*, 1968)	*k* = 0→6
*T*_min_ = 0.908, *T*_max_ = 0.969	*l* = −18→18
1946 measured reflections	3 standard reflections every 200 reflections
1852 independent reflections	intensity decay: 9%

### Refinement

**Table:**

Refinement on *F*^2^	Primary atom site location: structure-invariant direct methods
Least-squares matrix: full	Secondary atom site location: difference Fourier map
*R*[*F*^2^ > 2σ(*F*^2^)] = 0.041	Hydrogen site location: inferred from neighbouring sites
*wR*(*F*^2^) = 0.110	H-atom parameters constrained
*S* = 1.06	*w* = 1/[σ^2^(*F*_o_^2^) + (0.0521*P*)^2^ + 0.3815*P*] where *P* = (*F*_o_^2^ + 2*F*_c_^2^)/3
1852 reflections	(Δ/σ)_max_ < 0.001
128 parameters	Δρ_max_ = 0.20 e Å^−^^3^
0 restraints	Δρ_min_ = −0.26 e Å^−^^3^

### Special details

**Table:**

Geometry. All esds (except the esd in the dihedral angle between two l.s. planes) are estimated using the full covariance matrix. The cell esds are taken into account individually in the estimation of esds in distances, angles and torsion angles; correlations between esds in cell parameters are only used when they are defined by crystal symmetry. An approximate (isotropic) treatment of cell esds is used for estimating esds involving l.s. planes.
Refinement. Refinement of *F*^2^ against ALL reflections. The weighted *R*-factor *wR* and goodness of fit *S* are based on *F*^2^, conventional *R*-factors *R* are based on *F*, with *F* set to zero for negative *F*^2^. The threshold expression of *F*^2^ > σ(*F*^2^) is used only for calculating *R*-factors(gt) *etc*. and is not relevant to the choice of reflections for refinement. *R*-factors based on *F*^2^ are statistically about twice as large as those based on *F*, and *R*- factors based on ALL data will be even larger.

### Fractional atomic coordinates and isotropic or equivalent isotropic displacement parameters (Å^2^)

**Table:**

	*x*	*y*	*z*	*U*_iso_*/*U*_eq_	
S1	0.51511 (6)	1.47619 (10)	0.35880 (3)	0.0442 (2)	
O1	0.85492 (14)	0.6407 (3)	0.83956 (9)	0.0496 (5)	
N1	0.66878 (15)	1.0213 (3)	0.50804 (11)	0.0369 (4)	
N2	0.60984 (16)	1.2149 (3)	0.47967 (11)	0.0407 (5)	
H2	0.5936	1.3268	0.5155	0.049*	
N3	0.59915 (18)	1.0451 (4)	0.34546 (12)	0.0497 (5)	
H3A	0.6324	0.9237	0.3674	0.060*	
H3B	0.5800	1.0463	0.2910	0.060*	
C1	0.9085 (2)	0.4449 (6)	0.88050 (16)	0.0614 (8)	
H1A	0.9862	0.4377	0.8627	0.092*	
H1B	0.8706	0.3024	0.8634	0.092*	
H1C	0.9049	0.4624	0.9430	0.092*	
C2	0.84356 (18)	0.6327 (4)	0.75008 (14)	0.0383 (5)	
C3	0.77906 (17)	0.8120 (4)	0.71427 (13)	0.0366 (5)	
H3	0.7479	0.9264	0.7505	0.044*	
C4	0.76070 (17)	0.8221 (4)	0.62447 (13)	0.0350 (5)	
C5	0.8097 (2)	0.6510 (4)	0.57024 (14)	0.0428 (6)	
H5	0.7982	0.6566	0.5099	0.051*	
C6	0.8741 (2)	0.4764 (4)	0.60621 (16)	0.0503 (6)	
H6	0.9071	0.3642	0.5700	0.060*	
C7	0.8914 (2)	0.4638 (4)	0.69715 (16)	0.0472 (6)	
H7	0.9346	0.3430	0.7214	0.057*	
C8	0.69256 (18)	1.0136 (4)	0.58932 (13)	0.0379 (5)	
H8	0.6663	1.1311	0.6265	0.045*	
C9	0.57769 (17)	1.2279 (4)	0.39499 (13)	0.0332 (5)	

### Atomic displacement parameters (Å^2^)

**Table:**

	*U*^11^	*U*^22^	*U*^33^	*U*^12^	*U*^13^	*U*^23^
S1	0.0643 (4)	0.0374 (3)	0.0309 (3)	0.0090 (3)	−0.0081 (3)	0.0035 (2)
O1	0.0534 (10)	0.0621 (12)	0.0333 (8)	0.0106 (9)	−0.0062 (7)	0.0109 (8)
N1	0.0429 (10)	0.0363 (10)	0.0315 (9)	0.0058 (9)	−0.0045 (8)	0.0024 (8)
N2	0.0555 (12)	0.0378 (11)	0.0286 (9)	0.0118 (9)	−0.0091 (8)	−0.0020 (8)
N3	0.0732 (14)	0.0442 (12)	0.0315 (9)	0.0164 (11)	−0.0128 (9)	−0.0056 (9)
C1	0.0631 (16)	0.077 (2)	0.0444 (14)	0.0194 (15)	−0.0057 (12)	0.0225 (14)
C2	0.0335 (11)	0.0471 (14)	0.0343 (11)	−0.0031 (10)	−0.0041 (9)	0.0072 (10)
C3	0.0338 (11)	0.0429 (13)	0.0331 (11)	0.0020 (10)	0.0002 (9)	0.0024 (10)
C4	0.0331 (11)	0.0380 (12)	0.0340 (11)	−0.0035 (10)	−0.0043 (9)	0.0045 (10)
C5	0.0526 (14)	0.0413 (14)	0.0346 (11)	0.0013 (11)	−0.0061 (10)	−0.0020 (10)
C6	0.0622 (16)	0.0432 (14)	0.0454 (13)	0.0109 (12)	−0.0051 (12)	−0.0084 (11)
C7	0.0517 (14)	0.0401 (13)	0.0497 (14)	0.0076 (11)	−0.0091 (11)	0.0061 (11)
C8	0.0389 (11)	0.0439 (13)	0.0309 (11)	0.0039 (10)	−0.0012 (9)	0.0002 (10)
C9	0.0372 (11)	0.0357 (12)	0.0266 (10)	−0.0032 (10)	−0.0024 (8)	0.0015 (9)

### Geometric parameters (Å, °)

**Table:**

S1—C9	1.683 (2)	C2—C7	1.377 (3)
O1—C2	1.371 (2)	C2—C3	1.382 (3)
O1—C1	1.422 (3)	C3—C4	1.386 (3)
N1—C8	1.270 (3)	C3—H3	0.9300
N1—N2	1.370 (2)	C4—C5	1.402 (3)
N2—C9	1.346 (3)	C4—C8	1.454 (3)
N2—H2	0.8600	C5—C6	1.363 (3)
N3—C9	1.309 (3)	C5—H5	0.9300
N3—H3A	0.8600	C6—C7	1.402 (3)
N3—H3B	0.8600	C6—H6	0.9300
C1—H1A	0.9600	C7—H7	0.9300
C1—H1B	0.9600	C8—H8	0.9300
C1—H1C	0.9600		
			
C2—O1—C1	116.9 (2)	C4—C3—H3	119.9
C8—N1—N2	116.44 (18)	C3—C4—C5	119.4 (2)
C9—N2—N1	119.16 (18)	C3—C4—C8	118.6 (2)
C9—N2—H2	120.4	C5—C4—C8	122.00 (19)
N1—N2—H2	120.4	C6—C5—C4	119.8 (2)
C9—N3—H3A	120.0	C6—C5—H5	120.1
C9—N3—H3B	120.0	C4—C5—H5	120.1
H3A—N3—H3B	120.0	C5—C6—C7	120.9 (2)
O1—C1—H1A	109.5	C5—C6—H6	119.6
O1—C1—H1B	109.5	C7—C6—H6	119.6
H1A—C1—H1B	109.5	C2—C7—C6	119.1 (2)
O1—C1—H1C	109.5	C2—C7—H7	120.5
H1A—C1—H1C	109.5	C6—C7—H7	120.5
H1B—C1—H1C	109.5	N1—C8—C4	120.3 (2)
O1—C2—C7	124.6 (2)	N1—C8—H8	119.8
O1—C2—C3	114.8 (2)	C4—C8—H8	119.8
C7—C2—C3	120.6 (2)	N3—C9—N2	117.1 (2)
C2—C3—C4	120.2 (2)	N3—C9—S1	124.11 (16)
C2—C3—H3	119.9	N2—C9—S1	118.78 (16)

### Hydrogen-bond geometry (Å, °)

**Table:**

*D*—H···*A*	*D*—H	H···*A*	*D*···*A*	*D*—H···*A*
N2—H2···S1^i^	0.86	2.57	3.370 (2)	156
N3—H3B···S1^ii^	0.86	2.57	3.411 (2)	166
N3—H3A···N1	0.86	2.25	2.611 (3)	105

Symmetry codes: (i) −*x*+1, −*y*+3, −*z*+1; (ii) −*x*+1, *y*−1/2, −*z*+1/2.
